# Brain activation of semantic category-based grouping in multiple identity tracking task

**DOI:** 10.1371/journal.pone.0177709

**Published:** 2017-05-15

**Authors:** Liuqing Wei, Xuemin Zhang, Chuang Lyu, Siyuan Hu, Zhen Li

**Affiliations:** 1 School of Psychology, Beijing Key Lab of Applied Experimental Psychology, Beijing Normal University, Beijing, China; 2 State Key Lab of Cognitive Neuroscience and Learning & IDG/McGovern Institute for Brain Research, Beijing Normal University, Beijing, China; 3 Center for Collaboration and Innovation in Brain and Learning Sciences, Beijing Normal University, Beijing, China; 4 eMetric, LLC, San Antonio, Texas, United States of America; Chinese Academy of Sciences, CHINA

## Abstract

Using Multiple Identity Tracking task and the functional magnetic resonance imaging (fMRI) technology, the present study aimed to isolate and visualize the functional anatomy of neural systems involved in the semantic category-based grouping process. Three experiment conditions were selected and compared: the category-based targets grouping (TG) condition, the targets-distractors grouping (TDG) condition and the homogenous condition. In the TG condition, observers could utilize the categorical distinction between targets and distractors, to construct a uniform presentation of targets, that is, to form a group of the targets to facilitate tracking. In the TDG condition, half the targets and half the distractors belonged to the same category. Observers had to inhibit the grouping of targets and distractors in one category to complete tracking. In the homogenous condition, where targets and distractors consisted of the same objects, no grouping could be formed. The “TG-Homogenous” contrast (p<0.01) revealed the activation of the left fusiform and the pars triangularis of inferior frontal gyrus (IFG). The “TG-TDG” contrast only revealed the activation of the left anterior cingulate gyrus (ACC). The fusiform and IFG pars triangularis might participate in the representation of semantic knowledge, IFG pars triangularis might relate intensely with the classification of semantic categories. The ACC might be responsible for the initiation and maintenance of grouping representation.

## Introduction

People often need to localize a number of constantly moving objects and recognize their identities simultaneously in daily life situations, such as watching a football game on TV or on site, driving on a crowded street, and keeping an eye on their children who are running around on a noisy playground. The ability of humans to track and identify moving objects has typically been studied using Multiple Identity Tracking (MIT) task [1,2], derived from Multiple Object Tracking task [3]. In MOT/MIT task, several objects are presented to the observers at the same time. A subset of objects are designated as targets by flashing several times or being surrounded by colored lines, while the others as the distractors. Then all the objects move randomly for a few seconds. Observers’ task is to track the targets during the motion and report their locations and identities after the motion stops. Unlike the MOT task where all objects are identical during motion, in MIT task, objects with unique surface features (such as colors, numbers, animals or human faces) are employed as tracking stimuli, in order to investigate the effect of unique surface properties or identities on attentive tracking [4,5,6,7,8,9,10,11,12,13].

Previous research of MOT task suggest that a group representation could be formed based on objects’ spatiotemporal properties, such as speed [14]; motion trajectory [15], common motion [16] and spatial symmetry [17]. When observers could segregate the targets from distractors based on their distinct spatiotemporal properties, for example, the different velocities of targets and distractors [14], they have a better tracking performance. On the contrary, when the spatiotemporal information bound targets and distractors together, for example, when targets and distractors shared a chasing relationship [16], observers’ tracking performance would be impaired. Similar findings are indicated in the MIT task. Observers could use the surface properties such as color, size, shape, or their combination to construct and update the target configuration (in the targets group condition) to promote tracking. And the feature-based grouping was automatic to some extent, even bonding targets and distractors into one group was contrary to the task demands, it would still happen during tracking [4,5,17].

Recently, Wei, Zhang, Lyu and Li [18] demonstrated that observers could use targets and distractors’ categorical distinction to facilitate tracking in MIT task. They found consistent inter-category facilitation effect while the categorical difference between targets and distractors were animals vs. furniture, animals vs. fruits and land mammals vs. birds. Specifically, observers’ tracking performances were much better when targets and distractors were different categories (the inter-category condition) than when they were unique objects from the same category (the intra-category condition) or when they consisted of the same objects (the homogenous condition). In light of the above findings, Wei et al. [18] claimed that a category-based grouping mechanism may exist during tracking when targets and distractors are categorically distinct. Observers organized the targets and distractors into separate groups according to their categorical difference. It is very likely that treating the targets of the same category as one unit could lower observers’ working memory load of targets and make the targets recovery strategy much easier[6,7,8,11].

However, the neural basis of the category-based grouping in MIT task was unknown. Even though few studies investigated the brain activation patterns of category-based grouping representation, a great amount of research has examined the neural representation of categorical information in the brain. These research initiates from the findings of category-specific semantic deficits. Patients with category-specific semantic deficits have disproportionate or selective impairments for one semantic category compared to other semantic categories [19,20,21,22,23,24,25,26]. Both neuropsychological studies focusing on brain damaged patients and functional neuroimaging studies focusing on healthy individuals suggest dissociable neural circuitry that are specialized for representing knowledge of different conceptual domains [27]. For example, the representing of animate objects mainly includes two regions in posterior temporal cortex: the lateral portion of the fusiform gyrus and pSTS (the posterior region of superior temporal sulcus). The lateral portion of fusiform gyrus mainly represents animate objects’ visual form and pSTS represents their motion. The representing of common tools includes the medial portion of the fusiform gyrus as well as pMTG (the posterior region of middle temporal gyrus), IPS (intraparietal sulcus) and VPMC (ventral premotor cortex), these regions are all within the left hemisphere and are assumed to represent tools’ visual form and properties of motion and manipulation [28,29]. Previous research about the representation of concepts or semantic knowledge in the brain provides theoretical basis for our study. In the present study, we also used animals and tools as two distinct categories to induce the grouping construction, but our goal was not to determine localized brain regions specialized for objects of different categories, but was to examine the neural systems involved in the category-based grouping process.

As mentioned above, the purpose of this study was to investigate the brain activation pattern during category-based grouping in MIT task. In order to isolate the blood oxygen-level dependent (BOLD) responses for grouping representation, we designed three conditions: the targets grouping (TG) condition, the targets-distractors grouping (TDG) condition and the homogenous condition. In the TG condition, the targets were selected from one category and the distractors from another one. The observers could organize the targets within one group and the distractors within another group by their distinct semantic categories. In the TDG condition, half the targets and half the distractors were chosen from one category while the other halves from another category. Observers might treat half the targets and half the distractors as one group as they belong to the same category. In the homogenous condition, targets and distractors were the same object and no grouping happened. These conditions are compared to provide evidence for the following claims. Through the contrast of “TG-Homogenous”, we aimed to reveal brain systems involved in grouping presentation based on conceptual processing including objects recognition and categories classification. Through the contrast of “TG-TDG”, we aimed to reveal the neural basis of category-based grouping as well as the inhibition of targets-distractors grouping.

## Materials and methods

### Participants

Observers were recruited from Beijing Normal University through posting online from September 2014 to January 2015. All observers in the fMRI study volunteered to participate. Eighteen healthy volunteers (9 females, age range 20–28 years), all had normal or corrected to normal vision, reported no history of neurological or psychiatric disorder. All observers provided informed written consent. The study was approved by the Institutional Review Board (Ethics Committee) of the State Key Laboratory of Cognitive Neuroscience and Learning, School of Brain and Cognitive Sciences at Beijing Normal University. All observers received payment for their time.

### Materials

Microsoft Visual Basic.NET (version 2013) was used to program the stimuli and the experiment program run on a Core i5 laptop. Stimuli were projected onto a translucent screen placed at the back of the magnet bore. Participants viewed the screen through a mirror at a distance of ~30 cm from the eyes. The moving objects were presented within a white grey (RGB (255,255,255)) rectangle that subtended 480 × 360 pixels (24.49°× 18.44°), with a dark grey border (0.12° width, RGB (64, 64, 64)). There was always a grey fixation (0.73°×0.73°) in the center of the rectangle. The stimuli were 45 images of animals and 60 images of tools, fitting to a square of 40 × 40 pixels (2.05° × 2.05°).

The initial speed of each object was 9.37°/s. The speed was changed within 5% at each frame, from 5°/s to 13.75°/s.

### Design and task

This experiment contained three conditions: targets grouping (TG), targets-distractors grouping (TDG) and homogenous condition (see Table 1). In the TG condition, the targets were four unique animals’ images and the distractors were four unique tools’ images, and vice versa. In the TDG condition, the targets consisted of two animals’ images and two tools’ images, while the distractors consisted of another two animals’ images and other two tools’ images. In the homogenous condition, the targets and distractors were the same one animal or tool image. According to findings from previous studies [18], the categorical distinction between targets and distractors could facilitate tracking in the TG condition, compared to the homogenous condition, indicating a category-based grouping mechanism during tracking. The TDG condition shared the same stimulus with TG condition but had two categories distributed equally in both the targets and distractors. In the TDG condition, it was conjectured that the categorical information would impair tracking as half of the targets and half of the distractors were grouped in the same category. Two contrasts with the imaging data were performed: TG vs. homogenous and TG vs. TDG.

**Table 1:** The identities of targets and distractors of three conditions.

Conditions	Targets	Distractors
**Targets Grouping (TG)**	Animal A, Animal B, Animal C, Animal D	Tool A, Tool B, Tool C, Tool D
	Tool A, Tool B, Tool C, Tool D	Animal A, Animal B, Animal C, Animal D
**Targets Distractors Grouping (TDG)**	Animal A, Animal B, Tool A, Tool B	Animal C, Animal D, Tool C, Tool D
**Homogenous**	Animal A, Animal A, Animal A, Animal A	Animal A, Animal A, Animal A, Animal A
	Tool A, Tool A, Tool A, Tool A	Tool A, Tool A, Tool A, Tool A

The A, B, C, D represented the pictures of animals or tools used as stimuli. The categories of targets and distractors were randomly assigned, although only one particular combination is shown here. Please see the text for more details.

**Fig 1:**
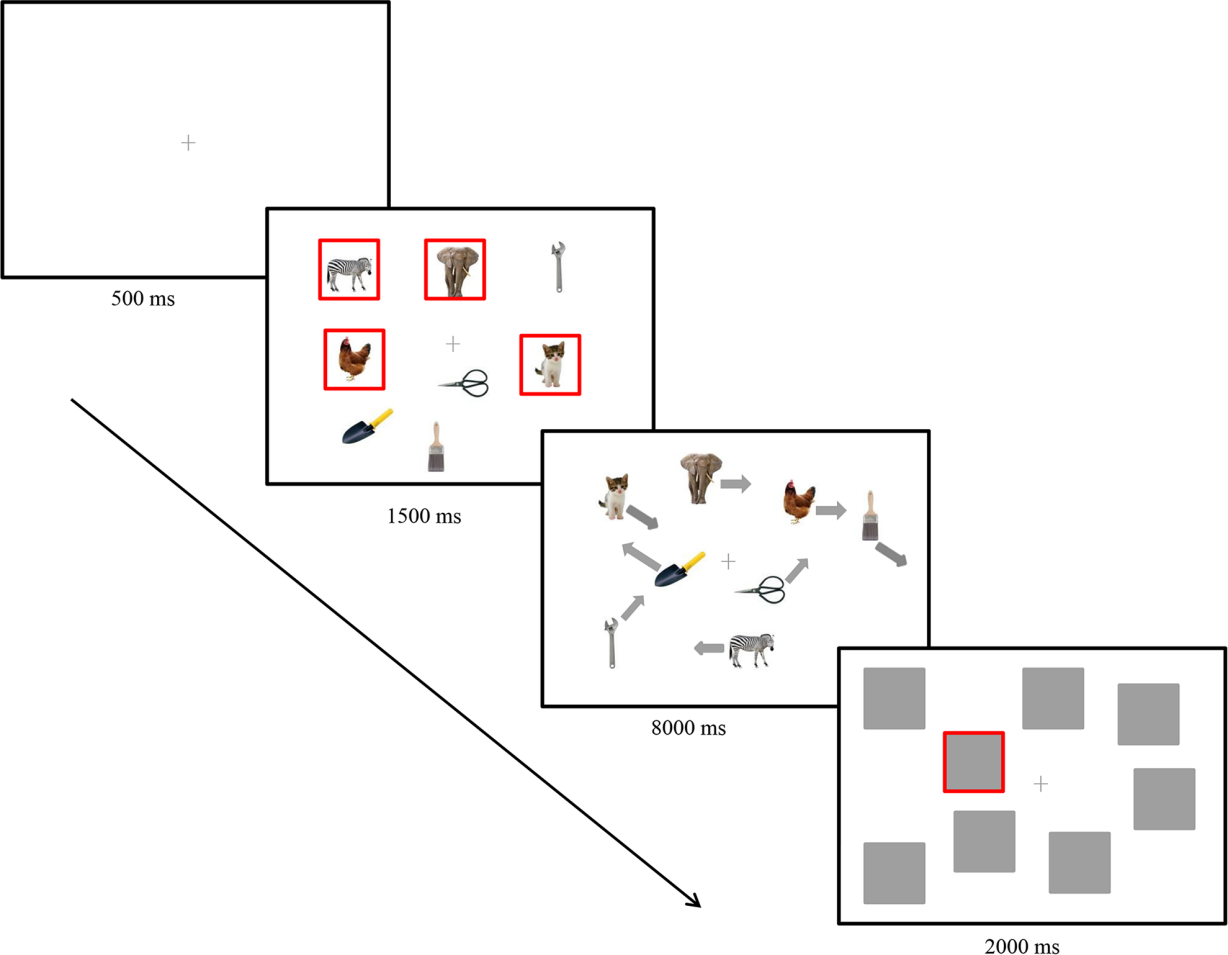
Sample illustrations of a trial in targets grouping condition.

At the start of each trial, a grey fixation cross was displayed for 500 ms in the center of the display. Observers were encouraged to maintain their fixation on the central cross during tracking. Then eight items were displayed, and four of them were surrounded by a red box (identified as targets) (1.47° width, RGB (255, 0, 0)), which lasted for 1500 ms. Then the red boxes disappeared and all of the pictures began to move randomly, which lasted for 8000 ms. Observers were instructed to track the four targets simultaneously during the motion. At the end of the motion, pictures were masked by grey squares subtended 40×40 pixels (2.05°×2.05°). One of the pictures was randomly surrounded by the red square. Observers were given 2000 ms to report whether it was one of the tracked targets. If the probed picture was one of the four targets, they were to press “1” using the left finger; if not, they were to press “2”. If no response was received within 2000 ms, the trial would be labeled “null”. (See Fig 1).

We employed a block design for the three conditions to indicate superior statistical power [30]. The TDG condition consisted of 30 trials, while both the TG and homogenous conditions consisted of 60 trials divided into two sub-conditions: the targets being animals and distractors being tools 30 trials, and the targets being tools and distractors being animals 30 trials in the TG condition (for the homogenous condition, the objects being animals 30 trials and being tools 30 trials). As a result, a total of 150 trials were organized into five blocks, each consisting of 30 trials. The length of each trial was 12 seconds. After each block, observers rested for 24 seconds. The whole experiment lasted approximately 32 minutes. Before scanning, observers were trained for 20 trials to ensure that they understood the task demands and well completed the task. The order effect of the five blocks was counterbalanced using a Latin square design. The correct answer of the trials was also balanced. In 50% of the trials, observers pressed the “1” key and in the other 50% of the trials they pressed “2”.

### Image acquisition

Imaging acquisition was performed on a 3T Scanner (Siemens, Trio Tim) in the Imaging Center for Brain Research of Beijing Normal University. A 12-channel head coil was used. A single-shot gradient-echo, EPI sequence was used to acquire the functional images (33 slices, interleaved slice order), with the following parameters: TR = 2000 ms, TE = 30 ms, FA = 90°, FOV = 200 × 200 mm^2^, matrix = 64 × 64, thickness = 4 mm, inter-slice gap = 0.6 mm (15%), voxel size = 3.125 × 3.125 × 4 mm^3^. An MPRAGE sequence was used to acquire high-resolution anatomical images of the entire brain with the following parameters: TR = 2300 ms, TE = 2.86 ms, FA = 9°, 144 slices, voxel size = 1 × 1 ×1.33 mm^3^.

### Statistical analysis of the imaging data

#### Image preprocessing and individual analyses

Adopting the same method as Lyu, Hu, Wei, Zhang and Talhelm [31], SPM12 software (Wellcome Department of Imaging Neuroscience, University College London, UK, http://www.fil.ion.ucl.ac.uk/spm) was used to preprocess and analyze the imaging data. The first six scans of each run were discarded from analysis to ensure that steady state tissue magnetization was reached. The remaining images were subject to preprocessing, including three-dimensional motion correction, spatial normalization into standard stereotaxic space (EPI template provided by the Montreal Neurologic Institute, MNI) with a resolution of 3 × 3 × 3 mm^3^, and spatially smoothing with a Gaussian kernel of FWHM of 6 mm.

The evoked hemodynamic responses to tracking (8000 ms) under three conditions, in trials with correct response, were modeled for each subject with a box-car function. Nuisance regressors consisting of the six head motion regressors from the SPM realignment procedure, the indexing (2000 ms) and response phase (2000 ms) of all trials, and the tracking phase in trails with no response or wrong responses were also added to the model. Two contrast analyses were conducted for each observer: TG vs. Homogenous and TG vs. TDG.

#### Group level analyses

As described in previous study [31], individual participants’ contrast maps were combined by a one-sample *t*-test for the two contrasts. The corrections for multiple comparisons (using AlphaSim correction) were confined within the whole-brain mask (size: 53,468 voxels) using the AlphaSim program in REST [32] (www.restfmri.net). When the statistical threshold was set at *p* = 0.01 and the voxels were edge connected, the thresholds of cluster size were 116 voxels for TG vs. Homogenous and 204 voxels for TG vs. TDG. Significance maps were then projected onto the inflated cortical surface of a standard brain provided by the BrainNet Viewer [33] (http://www.nitrc.org/projects/bnv/) program for display purposes.

## Results

### Behavioral results

The behavioral results are shown in Table 2. A repeated measures analysis of variance (ANOVA) indicated no significant main effect of the conditions (*F*(2, 34) = 0.156, *p* = 0.856, *η*^*2*^ = 0.009). This result differs from our previous study in that behavioral effect disappeared in the current study [18]. One possible explanation is that the objects’ speed of motion was much slower (the maximum speed was 13.75°/s) in the present experiment than that in the study of Wei et al. (the maximum speed was 24.1°/s)[18]. As a result, the tracking tasks become easier for observers in all the three conditions. Based on results in Table 2, we could infer that the difference of tracking difficulty between two conditions didn’t cause the brain activations in the contrasts below. However, since identity processing is involuntary in MIT task [6,8,12,13], objects’ identities are processed even when the processing consumes extra resources and impairs tracking performance [6,8]. In the TG condition, observers would automatically utilize the categorical distinction between targets and distractors to facilitate tracking, as revealed by Wei et al. [18]. In the TDG condition, objects’ category information would be processed and might interrupt tracking task. Therefore, even though the behavioral results in the present experiment showed no category-based grouping effect, based on previous findings, we conjecture that there still exists a target grouping in the TG condition and an inhibition of target-distractor grouping in the TDG condition. On the contrary, in the homogenous condition where targets and distractors were exactly the same object, observers had to monitor the targets’ trajectories of motion to complete the task.

**Table 2:** The average accuracies of three conditions (*Mean±SD*).

	TG	TDG	Homogenous
**Accuracy**	89.54%±4.88%	90.32%±6.29%	90.37%±7.13%

### Group activation maps

**Fig 2:**
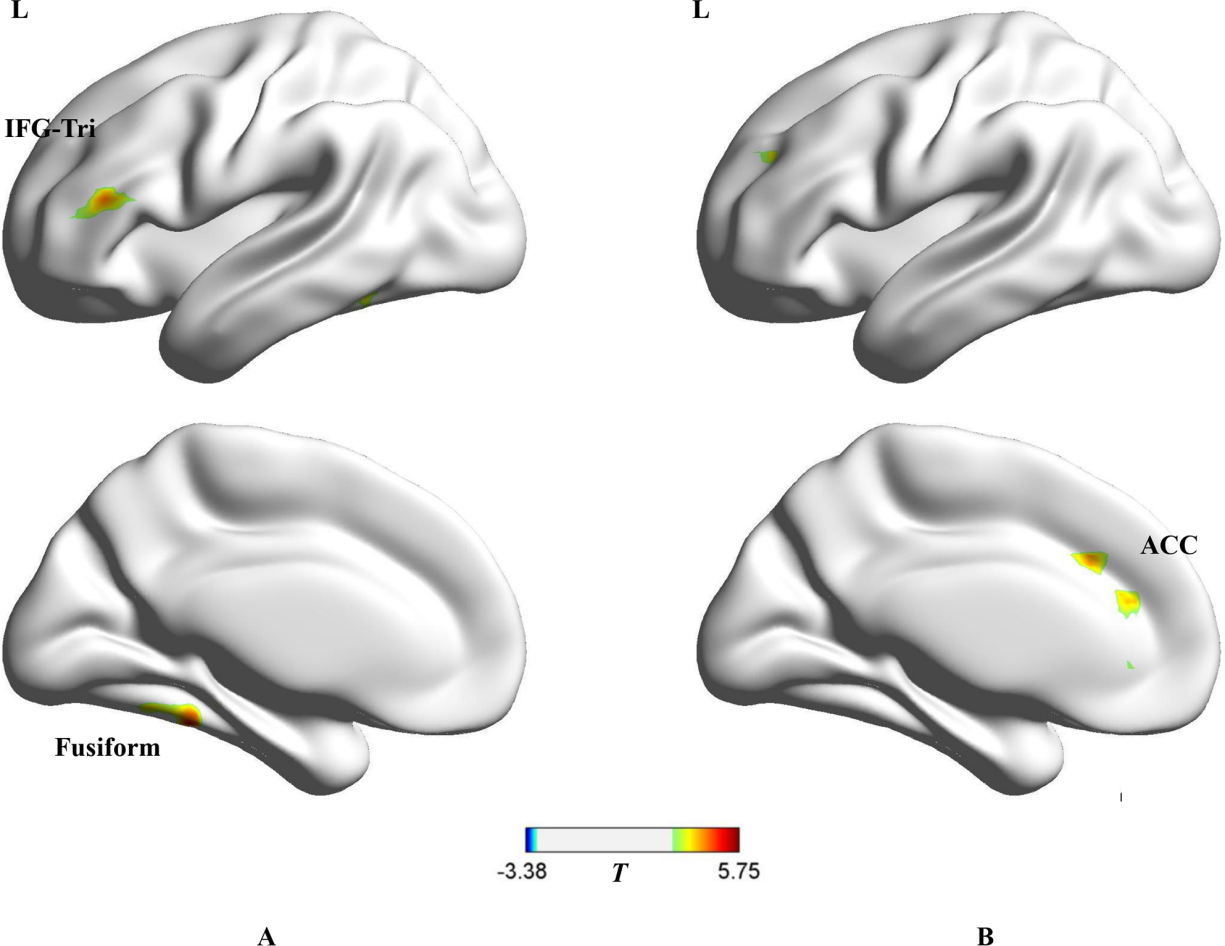
**A, TG condition vs. homogenous condition (*p* < 0.01, AlphaSim corrected).** The red shows the regions that are more active in the TG condition where observers could group the targets together based on the categorical distinction, as opposed to the homogenous condition where they localized the targets by tracking the trajectories of motion. **B, TG condition vs. TDG condition (*p* < 0.01, AlphaSim corrected).** The red shows the regions that are more active in the TG condition as opposed to in the TDG condition where they inhibited the targets-distractors grouping from impairing tracking.

Table 3, Fig 2A and 2B show the areas of activations in the “TG-Homogenous” and “TG-TDG” contrasts. The “TG-Homogenous” contrast reveals which brain areas were more active when observers could group the targets together and segregate them from the distractors based on their categorical distinction during tracking as opposed to localizing the targets only by tracking their trajectories of motion. Results in Table 3 and Fig 2A provide evidence that greater activity appears in left fusiform and left pars triangularis in the inferior frontal gyrus (IFG).

**Table 3:** The brain regions of activations in “TG-Homogenous” and “TG-TDG” contrasts.

Region	Lat.	BA	*x*	*y*	*z*	Vox.	*T*
**I. TG > homogenous**							
**fusiform gyrus**	L	37,20	-33	-42	-21	160	5.4731
**inferior frontal gyrus (pars triangularis)**	L	46	-42	33	15	140	4.6456
**II.TG > TDG**							
**anterior cingulate**	L	32,24	-27	24	30	335	5.7481

The “TG-TDG” contrast reveals the brain areas of activations when observers construct the grouping representation of targets to facilitate tracking as opposed to inhibiting the targets-distractors grouping from impairing tracking. From Table 3 and Fig 2B, it is indicated that this contrast had more activity in left anterior cingulate cortex (ACC).

## Discussion

Previous research revealed that grouping representation based on objects’ spatiotemporal properties and surface features existed in MOT/MIT task. Recently, Wei et al. [18] indicated that observers could utilize the categorical distinction between targets and distractors to facilitate tracking through the grouping of targets. Our goal in the present study was to localize the brain regions associated with the semantic category-based grouping in MIT task. In order to induce a category-based grouping effect, we chose animals and tools as two categories of tracking stimuli, which have been widely used to obtain category-specific neural responses in ventral and lateral occipital-temporal cortex [27,28,34]. We designed three conditions: TG, TDG and homogenous and performed two contrasts: “TG-Homogenous” and “TG-TDG”, with the imaging data.

The “TG-Homogenous” contrast revealed the activation of the left fusiform and the pars triangularis of inferior frontal gyrus. In a meta-analysis study of the category-specific neural processing for naming pictures of animals and naming pictures of tools, researchers demonstrated that the left fusiform presented greater activation in the contrasts of naming animals relative to baseline tasks versus naming tools relative to baseline tasks [34]. Specifically, animal pictures produced enhanced effects in the lateral posterior fusiform gyrus, and tool pictures elicited differential neural responses in the medial posterior fusiform gyrus [27,35]. The fusiform gyrus might participate in the processing of multiple types of stimulus properties [36,37] and shared features among objects [38]. In the TG condition, observers needed to process and retrieve the knowledge of eight objects (four animals and four tools). On the contrary, they only needed to process one object in the homogenous condition. The representation of object concepts was more demanding in the TG condition than in the homogenous condition. This might explain why the fusiform gyrus intensely associated with semantic processing was activated in this contrast.

The “TG-Homogenous” contrast also revealed the activation of the left pars triangularis of IFG. Previous study suggested that the activation of IFG, which was responsive to distractors’ identity switching in MIT task, was related to attention control [31]. Moreover, the pars triangularis was thought to fulfill a critical role in retrieving and manipulating semantic representations stored elsewhere in the brain [39,40]. It has also been involved in classifying objects into different categories [41]. In the TG condition, based on evidence from a previous behavioral study [18], observers could utilize the categorical distinction between targets and distractors to facilitate tracking in MIT task. However, in the homogenous condition, no categorical information was involved in tracking. Therefore, the activation of the left pars triangularis in the current contrast suggests that it may participate in retrieving the semantic representations of objects and classifying them.

As one of two prefrontal regions, ACC is particularly important in executive control. Its activations were consistently found for stimulus-response compatibility (Stroop), working memory, semantic generation, and episodic memory tasks. The ACC had three main functions: initiation, inhibitory, and motor. The initiation view refers to the anterior cingulate involved in the attentional processes to initiate behavior [42]; the inhibitory view proposes that the anterior cingulate participates in suppressing inappropriate behaviors or responses [43]. The initiation and inhibition functions of ACC are not incompatible: it can play a role in both initiating appropriate responses and suppressing inappropriate ones [44,45]. In the TG condition, the grouping of objects belonging to the same category was consistent with the task demands and could promote observers’ tracking performance. However, in the TDG condition, the grouping of objects based on their conceptual category was contrary to task demands and could impair tracking. As a result, observers might create the group of targets or the group of distractors respectively in the TG condition. The group formation might be automatic to some extent. However, in the TDG condition, in order to fulfill the tracking task, observers might intentionally inhibit the grouping of objects of the same category, that is, to avoid grouping of targets and distractors. Combined with the difference of TG and TDG conditions, the robust activation of ACC suggests that this cortical region may be related with the initiation and maintenance of the category-based targets grouping in the TG condition, or the suppression of targets-distractors grouping in the TDG condition. Since ACC was significantly more active in the TG condition than in the TDG condition, we inferred that the main role of ACC in the present study was the initiation and maintenance of targets grouping. The reason why the other brain areas were not activated in the TG-TDG contrast might be that both the TG and TDG conditions contained eight objects. The only difference between the two conditions was the distribution of objects in the targets and distractors. Therefore, much of the cerebral activity associated with low-level stimulus encoding, instructional set, and response output was controlled. Differences between TG and TDG conditions related directly to the grouping of targets (TG condition) and the inhibition of grouping of targets and distractors (TDG condition).

As why there was no common regions being revealed in the two contrasts, the reason might be that the TG-Homogenous contrast was not evident enough to isolate the brain regions related to grouping representation. Even though the grouping of targets in the TG condition was evident, weak targets grouping might also exist in the homogenous condition. Previous investigations of MOT task have shown that tracking performance was improved when observers employed the strategy of mentally grouping the identical targets into a single polygon and tracking the virtual object as a whole [14]. Eye movements’ studies have also demonstrated that observers tend to keep their gaze on the center of the targets during tracking, which suggests that observers may represent the targets as a group and help differentiate them from the distractors [46,47,48,49,50]. Therefore, the TG-Homogenous contrast might subtract out the grouping effect and make the corresponding brain regions hard to be revealed. Opposite of the homogenous condition, the targets grouping in the TDG condition was difficult and the targets-distractors grouping needed to be suppressed to meet the task demands. As a result, the TG-TDG contrast was evident enough to reveal the neural basis of category-based grouping.

Different from the previous research focused on the brain regions of category specificity, this study selected animals and tools as two typical categories to promote observers to construct a group of targets based on the categorical distinction between targets and distractors. The current study mainly investigated the neural basis of category-based grouping in MIT task. Based on the results of two contrasts, we inferred that the neural processing of category-based grouping in MIT task while using animals and tools as two respective categories was as following: at the first step, the semantic knowledge or conceptual representation might be processed and held in the left fusiform, then the objects might be classified into two semantic categories in the left pars triangularis. The conceptual representation and category classification formed the basis of grouping construction. The ACC might mainly participate in the initiation and maintenance of category-based grouping representation, by avoiding the disruption of distractors.

However, the current study is not without limitation. First, the tracking period was relatively short (8 s) in MIT task. In further studies, longer tracking period can be adopted to obtain more reliable results. Second, the behavioral effects of the current study were not significant, which might be due to the slow motion speed. In further studies, we will increase the motion speed to see if there exists significant behavioral effect and whether the present finding can be replicated.

In conclusion, according to the findings of the present study, the brain regions directly related to the category-based grouping effect in MIT task might be the left fusiform, the left pars triangularis of IFG and the left ACC. The fusiform and pars triangularis might mainly participate in the conceptual representation and categorical classification of the objects in MIT task. The ACC might be mainly responsible for the construction and maintenance of category-based grouping.

## Supporting information

S1 File The behavioral dataset of three conditions and imaging dataset of two contrasts. (ZIP)
